# Sarcoid reaction associated with Merkel cell carcinoma revealed by fluorodeoxyglucose positron emission tomography: a case report

**DOI:** 10.1186/1752-1947-5-282

**Published:** 2011-07-05

**Authors:** Yuko Higashi, Kentaro Mera, Mitsuyoshi Shimokawa, Mitsuhiro Hisadome, Atsunori Baba, Shigeto Matsushita, Masakazu Yanagi, Takuro Kanekura

**Affiliations:** 1 Department of Dermatology, Kagoshima University Graduate School of Medical and Dental Sciences, Kagoshima, Japan; 2 Surgical Oncology, Kagoshima University Graduate School of Medical and Dental Sciences, Kagoshima, Japan

## Abstract

### Introduction

Although the association between cancer and sarcoidosis or sarcoid reaction is known, sarcoid reaction associated with Merkel cell carcinoma is rare.

### Case presentation

We report the case of a 57-year-old Japanese woman with Merkel cell carcinoma in the inguinal area associated with sarcoid reaction. Fluorodeoxyglucose positron emission tomography demonstrated elevated fluorodeoxyglucose uptake by mediastinal lymph nodes and at the carcinoma site. Histopathologically, the mediastinal lymph nodes contained no Merkel cell carcinoma components. Sarcoid lesions were identified. Systemic examinations returned no sarcoidosis-specific findings.

### Conclusion

Fluorodeoxyglucose positron emission tomographic scans can be used to assess neoplastic lesions and depict sarcoidosis. Sarcoid reactions must be considered in the interpretation of fluorodeoxyglucose positron emission tomographic scans.

## Introduction

Sarcoidosis, a common systemic disorder of unknown etiology, is characterized by the formation of non-caseating epithelioid cell granulomas. The lungs, lymph nodes, liver, spleen, skin, eyes, small bones of the hands and feet and the salivary glands are most often affected [1]. A diagnosis of sarcoid reaction is made when localized epithelioid granulomas are found without signs of systemic sarcoidosis. They are attributable to infections, foreign materials, gastrointestinal diseases and malignant tumors [1]. The reported incidence of sarcoid reactions in carcinoma is 4.4%; in squamous cell carcinoma of the skin and mucous membranes, it is 13.0% [2]. Merkel cell carcinoma is a rare and aggressive skin cancer that is thought to arise from cutaneous Merkel cells which are neuroendocrine cells [3]. We present the case of a patient with Merkel cell carcinoma associated with sarcoid reaction.

## Case presentation

**Figure 1:**
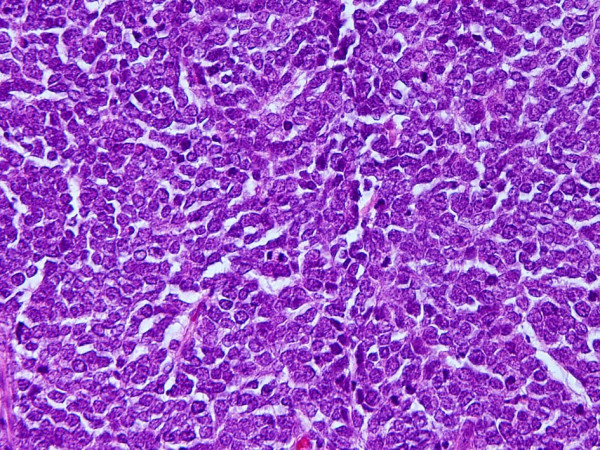
**Photomicrograph showing small to intermediate-sized cells with scant cytoplasm and regular nuclei with dusty chromatin**. No nucleoli are visible (hematoxylin and eosin stain; original magnification, ×400).

**Figure 2:**
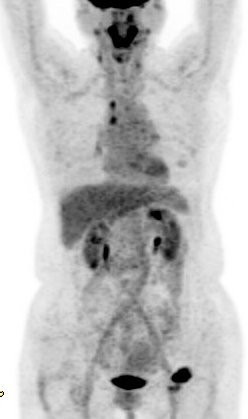
**Fluorodeoxyglucose (FDG) positron emission tomographic scan showing areas of FDG accumulation in the mediastinum and left inguinal region**.

**Figure 3:**
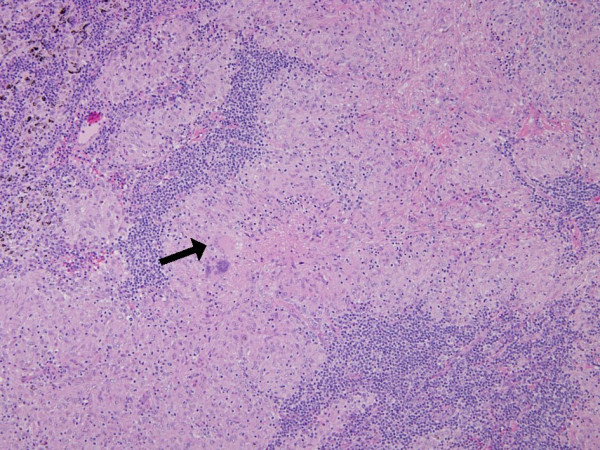
**Photomicrograph showing the pathological findings of non-caseating epithelioid cell granulomas with giant cells (arrow) (hematoxylin and eosin stain; original magnification, × 100)**.

A 57-year-old Japanese woman presented to our hospital with a painless, firm, palpable mass 3 cm in diameter of five months' duration in the left inguinal area. She was a non-smoker and had no particular respiratory symptoms. The resected inguinal mass was a dermal tumor consisting of small to intermediate-sized cells with scant cytoplasm and regular nuclei with dusty chromatin and no nucleoli (Figure 1). Immunohistochemically, the tumor cells were positive for cytokeratin 20 and negative for thyroid transcription factor 1. Our diagnosis was Merkel cell carcinoma. Contrast-enhanced computed tomographic (CT) scans showed right paratracheal and right tracheobronchial lymphadenopathies 1 cm in diameter, suggesting metastatic lesions. No lung parenchymal abnormality was found. A fluorodeoxyglucose positron emission tomographic (FDG-PET) study (Figure 2) of her mediastinal adenopathy revealed increased metabolic activity. The standardized uptake value was 6.1, corresponding to the nodal distribution seen on her CT scan. The sentinel lymph node in her left groin was excised and the mediastinal lymph nodes were dissected by performing video-assisted thoracic surgery. Her Merkel cell carcinoma was found to have metastasized to the superficial inguinal lymph node. Pathological examination of the mediastinal lymph node revealed a non-caseating epithelioid cell granuloma with giant cells. No tumor cells were identified. These findings were suggestive of sarcoidosis or sarcoid reaction (Figure 3). Her serum angiotensin-converting enzyme level was 3.5 U/L (normal 7 U/L to 25 U/L). No ocular manifestations were observed. Cardiac ultrasonography was within the normal range without an anomalous cardiac rhythm. The patient is being followed every three months and is free of relapse two years after the initial diagnosis.

## Conclusion

The association of sarcoid reaction with Merkel cell carcinoma is rare. Our literature review uncovered a case of an 84-year-old woman with Merkel cell carcinoma and chronic sarcoidosis [4]. Her carcinoma was diagnosed nine years after sarcoidosis, and ductal breast carcinoma was diagnosed six months before Merkel cell carcinoma. As the pathogenesis of sarcoidosis involves immune dysfunction [4], her two different malignant tumors may be attributable to disturbances in the immune system, which play an important role in immune surveillance. A longitudinal study adjusted for age, sex and smoking history showed a significantly higher incidence of cancer in patients with sarcoidosis than in the general population [5]. Sarcoidosis can develop after cancer, and in some patients there is an association between the administration of anti-neoplastic drugs and the appearance of sarcoidosis [6-8]. Although our patient manifested sarcoid reaction rather than sarcoidosis, it remains unknown whether sarcoid reaction in the presence of malignancy is different from systemic sarcoidosis involvement in the etiology of cancer.

Merkel cell carcinoma, composed of small, round blue cells, should be distinguished from small cell lung carcinoma, which has a similar pathological appearance. Their differentiation is possible because antibodies to cytokeratin 20 and thyroid transcription factor 1 are specific to Merkel cell carcinoma and small cell lung carcinoma, respectively [9].

In our patient, the immunohistochemical findings regarding the inguinal mass and the absence of tumor cells in the mediastinal lymph node led to a diagnosis of Merkel cell carcinoma arising in the inguinal region. Her sarcoid reaction was mediastinal. Although sarcoid reactions are most commonly observed in cancer-draining lymph nodes, they can occur in non-regional tissues [2,6]. They are thought to be attributable to soluble antigenic or granulomagenic factors derived from tumor cells and include antigen-antibody complexes, peptides and modified tumor cells [2]. Our observation that the sarcoid reaction in our patient occurred at distant tissue sites supports the involvement of soluble factors.

FDG-PET, widely used to assess neoplastic lesions, depicts the glucose avidity of tissues. Although FDG-PET images reflect the different utilization of glucose by normal and malignant tissues, they fail to differentiate malignancy from inflammation reliably. Increased glucose uptake has been reported in patients with benign disorders [10,11], and elevated FDG uptake has been observed in patients with sarcoidosis. Brudin *et al*. [12] proposed that FDG-PET images reflect disease activity and the distribution of sarcoidosis. In cancer patients with sarcoidosis [13,14] or sarcoid reaction [12], FDG-PET has shown lesions that mimicked lymph node metastases. Kaira *et al*. [16] reported that the use of L-[3-^18^F]-α-methyltyrosine PET (^18^F-FMT PET) in combination with FDG-PET can distinguish sarcoidosis from malignancy. Sarcoid reaction must be considered in the evaluation of cancer metastasis, and the acquisition of ^18^F-FMT PET scans is desirable.

## Consent

Written informed consent was obtained from the patient for publication of this case report and any accompanying images. A copy of the written consent is available for review by the Editor-in-Chief of this journal.

## Competing interests

The authors declare that they have no competing interests.

## Authors' contributions

YH drafted the manuscript and reviewed the literature. KM, MS, MH and AB obtained informed consent from the patient, assisted in data collection and coordinated and helped to draft the manuscript. SM and MY carried out the patient's surgery and revised the manuscript. TK was responsible for the diagnosis, patient management and review. All authors read and approved the final manuscript.
